# Exposure to Salinity and Light Spectra Regulates Glucosinolates, Phenolics, and Antioxidant Capacity of *Brassica carinata* L. Microgreens

**DOI:** 10.3390/antiox10081183

**Published:** 2021-07-26

**Authors:** Sylvia Maina, Da Hye Ryu, Jwa Yeong Cho, Da Seul Jung, Jai-Eok Park, Chu Won Nho, Gaymary Bakari, Gerald Misinzo, Je Hyeong Jung, Seung-Hoon Yang, Ho-Youn Kim

**Affiliations:** 1 Smart Farm Research Center, Korea Institute of Science and Technology (KIST), Gangneung 25451, Korea; wairimusylvia@kist.re.kr (S.M.); dahye0507@kist.re.kr (D.H.R.); chocho7023@kist.re.kr (J.Y.C.); 118521@kist.re.kr (D.S.J.); j-park@kist.re.kr (J.-E.P.); cwnho@kist.re.kr (C.W.N.); jhjung@kist.re.kr (J.H.J.); 2 SACIDS Foundation for One Health, Sokoine University of Agriculture, Morogoro 25523, Tanzania; gaymary.bakari@sua.ac.tz (G.B.); gerald.misinzo@sacids.org (G.M.); 3 Division of Bio-Medical Science and Technology, KIST School, University of Science and Technology (UST), Daejeon 34113, Korea; 4 Department of Medical Biotechnology, College of Life Science and Biotechnology, Dongguk University, Seoul 04620, Korea; shyang@dongguk.edu

**Keywords:** *Brassicaceae*, light wavelength, bioactive compounds, reactive oxygen species (ROS), oxidative stress, antioxidant enzymes, antioxidant proteins

## Abstract

The effect of salt treatment on *Brassica carinata* (BC) microgreens grown under different light wavelengths on glucosinolates (GLs) and phenolic compounds were evaluated. Quantifiable GLs were identified using ultra-high performance-quadrupole time of flight mass spectrometry. Extracts’ ability to activate antioxidant enzymes (superoxide dismutase (SOD) and catalase (CAT)) was evaluated on human colorectal carcinoma cells (HCT116). Furthermore, BC compounds’ ability to activate expression of nuclear transcription factor-erythroid 2 related factor (Nrf2) and heme-oxygenase-1 (HO-1) proteins was examined using specific antibodies on HCT116 cells. Sinigrin (SIN) was the abundant GLs of the six compounds identified and its content together with total aliphatic GLs increased in saline conditions. Fluorescent (FL) and blue plus red (B1R1) lights were identified as stable cultivation conditions for microgreens, promoting biomass and glucobrassicin contents, whereas other identified individual and total indole GLs behaved differently in saline and non-saline environments. Blue light-emitting diodes and FL light in saline treatments mostly enhanced SIN, phenolics and antioxidant activities. The increased SOD and CAT activities render the BC microgreens suitable for lowering oxidative stress. Additionally, activation of Nrf2, and HO-1 protein expression by the GLs rich extracts, demonstrate their potential to treat and prevent oxidative stress and inflammatory disorders. Therefore, effective salt treatments and light exposure to BC microgreens present an opportunity for targeted regulation of growth and accumulation of bioactive metabolites.

## 1. Introduction

Members of the family *Brassicaceae* have recently gained interest as nutraceutical foods and as a source of natural bioactive compounds, including phenolics and glucosinolates (GLs) [1]. Among these plants is Ethiopian mustard (*Brassica carinata* A. Braun), an orphan crop that originates from the highlands of Ethiopia, where it is known as “Gomenzer” (Yehabesha Gomen) and “Hamli Adri” in Amharic and Tigrigna languages [2]. *B. carinata* usefulness as an oilseed crop and as a vegetable has caused its cultivation to spread to other arid and semi areas of the world [3]. Also, the newly developed consumer-friendly varieties of Ethiopian mustard have caused increased acceptability of this nutritious vegetable [2].

The leaves and seeds of *B. carinata* are rich in nutrients, including proteins, carbohydrates, vitamins, and carotenoids [4,5]. Moreover, the vegetable has been reported to contain polyphenols and high contents of GLs, (such as sinigrin (SIN)) whose levels are approximated to be several folds higher compared to cabbage, broccoli, Chinese cabbage, and Korean leaf mustard [2,6]. These compounds owe most cruciferous vegetables their health-promoting potential with GLs hydrolysis products being associated with cancer prevention, antimicrobial, anti-inflammatory and polyphenols with antioxidant activities [7,8,9].

Polyphenols and GLs have been reported in several studies to be important natural antioxidants during oxidative stress to control the production of reactive oxidants such as reactive oxygen species (ROS) and reactive nitrogen species (RNS) [10]. The controlled production of the oxidants maintains their balanced concentration, which would otherwise cause them to nab cellular electrons resulting in chronic diseases such as diabetes, cancers and cardiovascular ailments [11,12]. Antioxidants also activate expression of phase II detoxifying proteins ((such as heme-oxygenase-1 (HO-1) through the activation of nuclear transcription factor-erythroid 2 related factor (Nrf2)); and antioxidant enzymes ((including superoxide dismutase (SOD), glutathione peroxidase (GPx) and catalase (CAT)). The antioxidant enzymes make it easier for the antioxidants to donate electrons and also, they assist in the recycling process by enabling reduction reactions of oxidized antioxidants [10]. Nrf2 pathway activation inhibits the progression of inflammation while expression of Nrf2 mediated antioxidant gene reduces the production of ROS [13].

In the majority of *Brassicaceae* species including kale, radish, and broccoli, higher contents of GLs and phenolic bioactive compounds are reported in juvenile stages of plants compared to mature plants [1,14,15], where the compounds decrease over time as a result of tissue expansion. The rapid changes in the compounds profiles during germination and early growth of vegetables make early harvesting a particularly relevant factor for maximizing the concentration of these desirable bioactive compounds [1,14]. This has contributed to the rising popularity of young vegetables (sprouts and microgreens), as a source of both health-promoting compounds and as functional foods [15,16,17].

During cultivation, elicitors possess the ability to induce changes that activate plant signaling pathways and enhance the production of specific phytochemicals [18,19]. Sodium chloride (NaCl) salt stress for instance causes physiological and biochemical perturbations in plants providing a strategy to increase GLs biosynthesis [20]. In particular, salt stress demonstrated to be effective in enhancing GLs in broccoli, kale, and radish [21] and thus has shown potential industrial applications [22,23].

Elicitation and production of healthier vegetables can be done stably in controlled environments, such as indoor vertical farm closed cultivation systems with regulated conditions of light irradiation, temperature, humidity, nutrients, and carbon dioxide [24,25,26]. Such farms are emerging as promising systems for the production of plant-derived medical ingredients and functional foods [27,28,29]. In these farming systems, light serves as an important factor due to its role in the plant’s development, growth, morphogenesis, and biosynthesis of pigment [30]. In particular, light-emitting diodes (LEDs) are mainly used because of their high energy conservation efficiency, wavelength specificity, and adjustable light quality and intensity [31]. The responses exhibited by plants on exposure to monochromatic and combined lights are associated with distinct activation of phytochromes and photoreceptors that induce metabolic changes [32] therefore, some LED wavelengths are used to accumulate specific compounds and nutrients of interest in plants [33]. For instance, red (R) and blue (B) LEDs have been used in *Brassicaceae* species to increase anthocyanin, carotenoids, GLs, lutein, vitamin, soluble sugar, soluble protein, and polyphenolic contents [25,34,35,36,37].

Despite the perceived health importance of *B. carinata* vegetables from the reported available phytochemicals, and their applicability in a closed cultivation system, to date, this scientific information is lacking. It is, therefore, necessary to investigate the occurrence of the bioactive compounds, the health benefits, and conditions for enhancing the growth and production of high quality and phytochemical-rich microgreens. Thus, this study aimed to identify GLs profiles, determine the phenolic compound contents and assess the biological activities of *B. carinata* microgreens treated with salt stress and cultivated under different light wavelengths in an indoor closed system.

## 2. Materials and Methods

### 2.1. Plant Materials and Growth Conditions

Seeds of *B. carinata* obtained from the Kenya Resource Centre for Indigenous Knowledge, National Museums of Kenya, were cultivated in a vertical indoor farming system built at the SMART u-FARM at the Korea Institute of Science and Technology (Gangneung, South Korea). Before sowing, the seeds were washed and placed in a tray (30 cm × 20 cm × 5 cm) with a mesh plate. Plants were cultivated at 18–26 °C and relative humidity of 50–80% under closed and controlled cultivation conditions. Plants were exposed to 200 ± 11 μmol/m^2^s of light intensity from fluorescent lamps (TL-D 18W/865; Philips, Amsterdam, The Netherlands) at 25 cm under a 14 h/10 h light/dark cycle for seven days after sowing (DAS) and then treated with elicitors. The plants were harvested at 14 DAS and their fresh biomass was weighed. The *B. carinata* microgreens were frozen in liquid nitrogen and stored at −80 °C before freeze-drying for five days to analyze chemical constituents and bioactivities of interest. The experiment was a randomized complete block design and was replicated thrice.

### 2.2. Elicitor Treatment and Sampling

After 7 DAS, *B. carinata* uniformly germinated plants were supplied with a nutrient solution and exposed to eight treatment of combinations of light wavelength and saline/non-saline solutions. The nutrient solution had an electrical conductivity of 1.2 ± 0.05 dS/m and contained 354 ppm N, 186 ppm P, 420 ppm K, 230 ppm Ca, 13 ppm Mg, and 83 ppm S (macronutrients), and 2.80 ppm Fe, 0.32 ppm B, 0.77 ppm Mn, 0.04 ppm Cu, 0.02 ppm Zn, and 0.02 ppm Mo (micronutrients). Fluorescent lamps (TL-D 18W/865; Philips Electronics, Seoul, South Korea) and LED lamps (KLB-40-2C; KAST Engineering, Gumi, South Korea) with a light intensity of 85 ± 11 μmol/m^2^s were used as artificial light sources at 30 cm. The light conditions included fluorescent (FL)- the control, B LED (440 nm + warm white), R LED (660 nm), and B plus R LED at a 1:1 ratio (B1R1) for seven days. Additionally, a 100 mM NaCl solution (this concentration was selected based on a literature review [23,38]) was added to the nutrient solution and supplied as an elicitor for three days before the plants were harvested. At 14 DAS, 30 microgreens were randomly selected, collected, and combined to form each of the three replicates used in further processing and analysis.

### 2.3. Sample Extraction and Desulfation of Standards

Extracts of desulfated GLs were prepared following previously published protocols [21,39], with minor modifications. Freeze-dried sample (0.05 g) was immersed in 70% methanol (1 mL) and heated at 90 °C in a heating block for 30 min. After cooling, the samples were centrifuged at 2063× *g*, 4 °C for 15 min and the extraction process was repeated one more time after which 1.2 mL of the supernatant was transferred to new Falcon tubes containing 0.1 mM glucotropaeolin (GTR) (20 μL) as an internal standard and reacted with 0.15 mL of lead acetate and barium acetate mixture (1:1, *v/v*), then centrifuged at 13,475× *g*, 4 °C for 5 min. One milliliter of the supernatant was loaded onto a mini-column packed with diethyl-aminoethyl Sephadex A-25 anion exchange resin, which had been pre-activated with 0.1 M sodium acetate and rinsed with deionized water twice. The loaded sample was reacted with 200 μL of 0.1% purified arylsulphatase (*Helix pomatia* Type H-1; Sigma-Aldrich, St. Louis, MO, USA) at room temperature for 16 h for desulfation. Desulfo-GLs (DS-GLs) were eluted with 0.5 mL of distilled water and filtered through a 0.2 μm polyvinylidene difluoride filter for analysis.

The GLs were analyzed using desulfated standards of SIN (CAS 3952-98-5), GTR (CAS 499-26-3), glucobrassicin (GBS) (CAS 143231-38-3) (Extrasynthese, Genay, France), and neoglucobrassicin (GNBS) (CAS 5187-84-8) (Extrasynthese, Genay, France). One milliliter of each standard (2 mM) was loaded in pre-activated diethyl-aminoethyl resin and reacted with arylsulphatase for conversion into their desulfated forms.

For the phenolic contents and biological assays, 0.1 g of freeze-dried sample was extracted with 70% ethanol (2 mL) at 40 °C for 15 min. The extracts were filtered and concentrated to dryness before re-dissolving in dimethyl sulfoxide solvent.

### 2.4. GL Analysis Using Ultra-High-Performance Liquid Chromatography (UHPLC)-Diode Array Detection (DAD)-Quadrupole Time-of-Flight (QTOF)-Mass Spectrometry (MS)

DS-GLs were analyzed using an Agilent 1290 UHPLC system (Agilent, Waldbronn, Germany) combined with a QTOF instrument (Bruker Daltonics, Bremen, Germany). Samples (20 μL) were injected into a YMC-Triart C18 ExRS column (150 × 2.0 mm; particle size, 1.9 μm) and separated with mobile phases consisting of water (solvent A) and acetonitrile (solvent B), each acidified with 0.2% (*v/v*) formic acid. The gradient system composition was as follows: solvent B was kept at 0% for 1 min, and its ratio increased to 15%. This condition was continued for 4 min then; the B ratio increased to 100% for 7 min. 100% solvent A was applied for 1 min for column rinsing. The auto-sampler temperature was set at 4 °C, and the column temperature was maintained at 50 °C. Negative electrospray ionization mode was used for all compounds. The drying gas temperature was set at 300 °C with a flow rate of 8.0 L/min, while the nebulizer gas pressure and capillary voltage were maintained at 2.4 bar and 4000 V, respectively. Ionization quadrupole ion energy and collision energy were set at 2 eV and 20 eV, respectively and, for screening, the detection mass range was set at 100–700 *m/z*. All DS-GLs were detected at a wavelength of 229 nm and analyzed by comparing retention times with the available standards including the internal standard (GTR) and their specific fragmentation patterns. Their contents were calculated using the relative response factor (RRF) as previously reported [40,41] relative to the desulfoglucotropaeolin. After calculating the contents using Equation (1), described in the standard protocol [42], the contents were expressed in (µmoles/100 g).

(1) $$\frac{\mathrm{Relative}\;\mathrm{peak}\;\mathrm{area}\;\mathrm{of}\;\mathrm{desulfoglucosinolate}}{\mathrm{Relative}\;\mathrm{peak}\;\mathrm{area}\;\mathrm{of}\;\mathrm{internal}\;\mathrm{standard}}\;\times \;\frac{\mathrm{µmoles}\;\mathrm{of}\;\mathrm{the}\;\mathrm{internal}\;\mathrm{standard}}{\mathrm{dry}\;\mathrm{weight}\;\mathrm{(g)}\;\mathrm{of}\;\mathrm{extracted}\;\mathrm{plant}\;\mathrm{material}}\;\times \;\mathrm{RRF}\;\mathrm{of}\;\mathrm{desulfoglucosinolate}$$

**Equation (1)**: Calculation of the content of each desulfoglucosinolate relative to the desulfoglucotropaeolin internal standard, expressed in µmoles/100 g of the sample dry weight.

### 2.5. Determination of the Total Phenolic Content (TPC)

#### Reagents and Chemicals

Folin-Ciocalteu reagent, gallic acid standard were obtained from Sigma-Aldrich Co. (St. Louis, MO, USA). Sodium carbonate was obtained from Fisher Scientific (Fair lawn, NJ, USA). Phenolic contents were determined by the Folin-Denis method, with some modification [43]. A 10-μL sample (4 mg/mL) was mixed with 2% Na_2_CO_3_ solvent (100 μL) in a 96-well plate that was then agitated for 3 min. Then, 10 μL of Folin–Ciocalteu reagent was added and the plate was incubated in the dark at room temperature for 30 min. The absorbance, at 750 nm was read using a multi detection microplate reader (Synergy HT; BioTek Instruments, Winooski, VT, USA). TPC was calculated based on a calibration curve determined using gallic acid as a standard and was expressed as mg gallic acid equivalents (GAE)/100 g of DW (dry weight).

### 2.6. Cell Culture, and Antioxidant Enzyme Superoxide Dismutase (SOD) and Catalase (CAT) Activity Assay

Human colon carcinoma cells (HCT116) were obtained from the ATCC (Manassas, VA, USA) and cultured in minimum essential media (Hyclone, Logan, UT, USA), supplemented with 10% fetal bovine serum (Thermo Fisher Scientific, Waltham, MA, USA) 1% penicillin-streptomycin mixture solution (Hyclone Logan, UT, USA) at 37 °C in a 5% CO_2_ atmosphere. The cells were seeded (5 × 10^4^ cells per well in a 24-well plate) and maintained for two days until their confluence reached 80%, the point at which media was removed. After removing the media, the cells were rinsed twice with phosphate-buffered saline (PBS; Hyclone Logan), and another media with or without 100 µg/mL extracts were added into the wells. The media was removed after 48 h, and the cells were washed twice with PBS. Cell SOD and CAT activity were evaluated using Abcam’s commercial colorimetric assay kits (Abcam, Cambridge, UK). In brief, the cells were lysed with lysis buffer available in the assay kits, and SOD and CAT activity were measured according to the manufacturer’s protocol.

### 2.7. Western Blotting of Nrf2 and HO-1

Cells treated with sample extracts were lysed using radioimmunoprecipitation assay buffer (RIPA buffer) (Thermo Fisher Scientific, Waltham, MA, USA) with a 1% protease inhibitor cocktail (Sigma-Aldrich, St. Louis, MO, USA). The protein concentrations in each lysate were quantified using the Bradford assay kit (Bio-Rad, Hercules, CA, USA). Proteins were denatured in sodium dodecyl sulfate (SDS) sample buffer separation with SDS polyacrylamide gel electrophoresis (SDS-PAGE). The proteins were then transferred to polyvinylidene fluoride (PVDF) membranes (Bio-Rad, Hercules, CA, USA) which were blocked with 3% bovine serum albumin in phosphate-buffered saline with tween solution. After 1 h, membranes were incubated with specific primary antibodies and horseradish peroxidase (HRP)-conjugated mouse and rabbit secondary antibodies. The antibodies against Nrf2 (Abcam, Cambridge, UK) and antibodies against β-actin and HO-1 (Santa Cruz Biotechnology, Dallas, TX, USA) were used. To visualize protein bands, ECL Western blotting detection kit (Thermo Fisher Scientific, Waltham, USA) and ImageQuant LAS-4000 (Fujifilm, Tokyo, Japan) were used. The quantification of protein bands was determined with ImageJ software (NIH, Bethesda, MD, USA).

### 2.8. Statistical Analysis

Data are expressed as the mean ± standard error. A comparison of means between two groups was done with the Student’s t-test while among groups the means were compared by one-way analysis of variance (ANOVA) with Duncan’s post-hoc tests in the SPSS software version 26. Principal component analysis (PCA) and orthogonal partial least squares discriminant analysis (OPLS-DA) was performed for UHPLC-QTOF-MS analysis results, total phenolic contents, antioxidant activity, and chemo-preventive ability, using the SIMCA program to understand the correlations between GLs a value of *p* < 0.05 was considered significant.

## 3. Results

### 3.1. Effects of LED Light and Salt Stress on Microgreens Biomass

**Figure 1:**
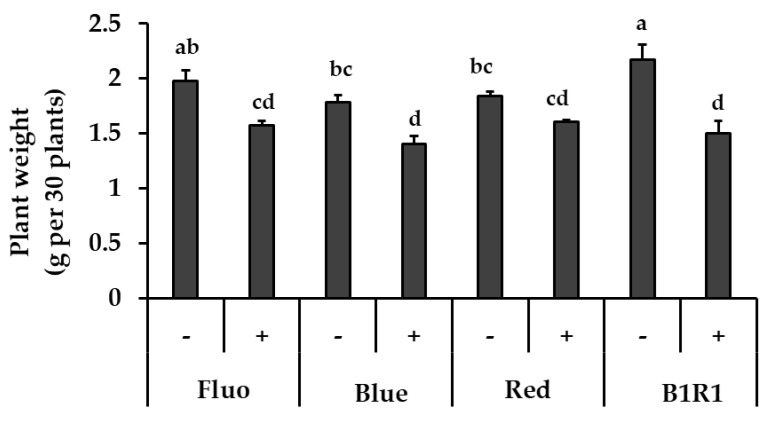
The effect of light, fluorescent (FL), blue, red, B1R1 (combination of blue plus red) on the biomass of microgreens cultivated in the absence (−) and presence (+) of salt, one week after treatments (*n* = 90). Data were expressed as mean ± standard error of three replicates. ANOVA analysis was done using Duncan’s method and different letters were used to show statistical significance (*p* < 0.05).

The exposure of the microgreens to various LEDs and salinity had a significant impact on their fresh weight (Figure 1). Salinity treatment generally produced microgreens with lower biomass; although these plants did not show any visible signs of stress. The microgreens under B1R1 LED had the highest biomass, 1.10 folds higher compared to microgreens exposed to the control FL light (which is the commonly used light during cultivation). Lower biomass was generally observed in microgreens treated with salinity under all the various light irradiations with some specific lowest significant values in microgreens exposed to B and B1R1 LED.

### 3.2. Total Phenolic Content (TPC)

**Figure 2:**
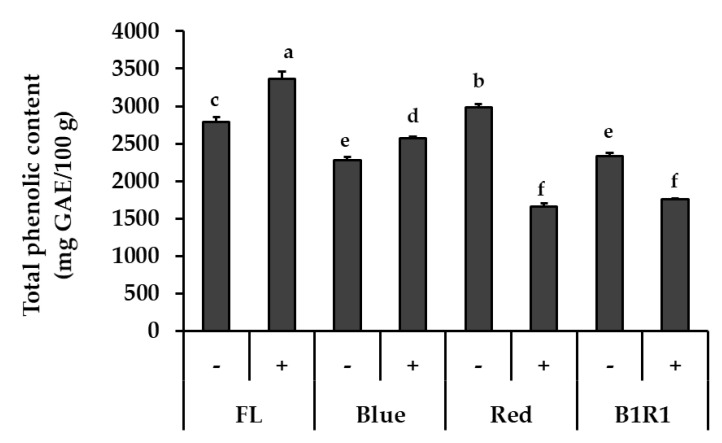
Total phenolic content of microgreens cultivated in the absence (−) and presence (+) of salt and exposed to fluorescent (FL), blue, red and combination of blue plus red (B1R1) light. Data were expressed as mean ± standard error (*n* = 3) in milligram(mg) gallic acid equivalent (GAE) per 100 g (g) of the extracts dry weight (DW). ANOVA analysis was done using Duncan’s method and different letters (a–f) were used to show statistical significance (*p* < 0.05).

Figure 2 shows the TPC of microgreens grown under various conditions. The most evident result was that the tendency of change in TPC was different depending on the light condition. The light wavelength, in particular, influenced these contents, with FL light exhibiting higher levels while R and B1R1 LED exhibited lower levels of TPC. While salinity increased TPC for microgreens under B LED at 1.13-fold, *p* = 0.035 in comparison to their counterparts in non-saline conditions, only TPC of microgreens cultivated in saline conditions and exposed to FL light increased significantly (1.19-fold, *p* = 0.017) in comparison to the control. Microgreens grown under R and B1R1 LED, on the other hand, showed a significant negative effect of salt treatments, with a decrease of 0.56-fold and 0.75-fold compared to salt-free groups, *p* = 0.004 and 0.003, respectively.

### 3.3. Identification of DS-GLs by UHPLC-QTOF-MS

The GLs identified in the *B. carinata* microgreen extracts are presented in Table 1 and Figure S1. Seven GLs, including the internal standard (GTR) and belonging to the aliphatic and indolic classes, were detected based on differences in their side-chain structures as illustrated in the cited papers. The aliphatic GLs (derived from methionine), included SIN; Rt = 5.51 min, C_10_H_17_NO_6_S) and gluconapoleiferin (GNL; Rt = 6.72 min, C_12_H_21_NO_10_S_2_), whereas the indolic GLs (derived from tryptophan) identified were 4-hydroxy glucobrassicin (HGBS; Rt = 7.17 min, C_16_H_20_N_2_O_10_S_2_), glucobrassicin (GBS; Rt = 9.33 min, C_16_H_20_N_2_O_9_S_2_), 4-methoxy glucobrassicin (MGBS; Rt = 11.25 min, C_17_H_22_N_2_O_10_S_2_), and neoglucobrassicin (GNBS; Rt = 12.44 min, C_17_H_22_N_2_O_10_S_2_) [44,45,46,47,48].

### 3.4. Quantification of Aliphatic GLs by UHPLC-DAD

**Figure 3:**
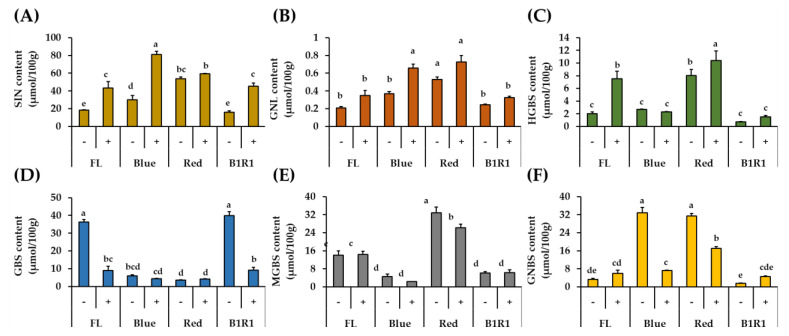
Quantification of DS-GLs (sinigrin (**A**), gluconapoleiferin (**B**), 4-hydroxy glucobrassicin (**C**), glucobrassicin (**D**), 4-methoxy glucobrassicin (**E**) and neoglucobrassicin (**F**)) in microgreens exposed to fluorescent (FL), blue, red and a combination of blue plus red (B1R1) lights and cultivated in the absence (−) and presence (+) of salt stress. Data were expressed as mean ± standard error (*n* = 3). ANOVA analysis was done using Duncan’s method and different letters on columns of each graph were used to show statistical significance (*p* < 0.05).

In this study, GLs compounds were quantified by comparing the desulfated compounds’ HPLC peak areas, retention times, and response factors relative to that of the internal standard GTR, which was included during the extraction process (Table 1 and Figure 3). The findings revealed distinct profiles of the identified GLs, as well as significant differences in their contents between treatments. When compared to indole group GLs, the total aliphatic GLs content was consistently high in all treatments, with specific values of SIN ranging from 16.15 μmol/100 g to 81.18 μmol/100 g and GNL ranging from 0.21 μmol/100 g to 0.73 μmol/100 g. These aliphatic GLs, both showed a similar pattern of increment in the salt treatments, regardless of the type of light irradiation and B LED specifically, significantly influenced their increase at 2.8 folds and 1.8-folds for SIN and GNL, respectively comparing to the contents of microgreen in the FL control group in absence of salt.

### 3.5. Quantification of Indole GLs by UHPLC-DAD 

The four detected indole GLs: GBS, HGBS, MGBS and GNBS, responded differently to salt treatment and LED exposure (Figure 3). The content of GBS, the parent GL, was negatively correlated with the contents of the other compounds (HGBS, *r* = −0.5421; MGBS, *r* = −0.2525; GNBS, *r* = −0.5627); and, except for HGBS, salinity did not favor the accumulation of any of the other indole GLs. The highest amounts of GBS were detected in microgreens grown in control and non-saline conditions of B1R1 LED, whereas its byproducts accumulated differently in microgreens exposed to B and R LED, with HGBS and MGBS being conspicuously elevated in microgreens exposed to R LED and GNBS being elevated in microgreens exposed to both R and B LED.

### 3.6. Anti-Oxidant Enzyme Activity

**Figure 4:**
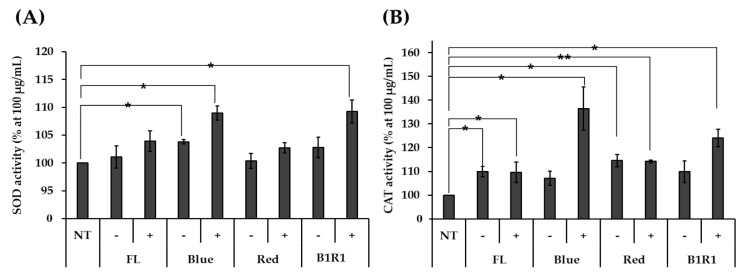
The activity of antioxidant enzymes superoxide dismutase (SOD (**A**)) and catalase (CAT (**B**)) was determined with various microgreen extracts at 100 μg/Ml using HCT116 human colorectal carcinoma cells. Extracts of microgreens exposed to fluorescent (FL), blue, red and blue plus red (B1R1) in the absence (−) or presence (+) of salt stress were treated to stressed cells while the control cells remained untreated. The experiment was conducted with three replications and data were expressed as mean ± standard error (*n* = 3). Activities were expressed as a percentage (%) compared to the non-treated (NT) group. Student t-test was conducted to see the difference between enzyme activity on untreated cells and cells treated with extracts from the various treatments. Extracts from microgreens exposed to B and B1R1 LED in the presence of salt exhibited higher activity than those from the microgreens cultivated in the control conditions. * = *p* < 0.05 and ** = *p* < 0.01 vs. the NT group.

The effect of the nontoxic microgreen extracts on the activities of SOD and CAT antioxidant enzymes was measured in HCT116 human colorectal carcinoma cells and compared against non-exposed cells. As shown in Figure 4A,B, stressed cells were treated with various microgreen extracts, which increased the activities of both enzymes, with microgreen extracts from saline treatments with higher overall activities than their counterparts. Specific high significantly different activities were observed in extracts of microgreens exposed to B and B1R1 LED, extracts with even higher activities than those from the control group. The microgreens from the saline treatment in R LED also showed significantly higher activity for CAT, as did the microgreens from the non-saline treatments in B LED for SOD, though their activity was not considerably higher than the activity of the control groups.

### 3.7. Effect of Microgreen Extracts on the Expression of Nrf2/HO-1 Pathway

**Figure 5:**
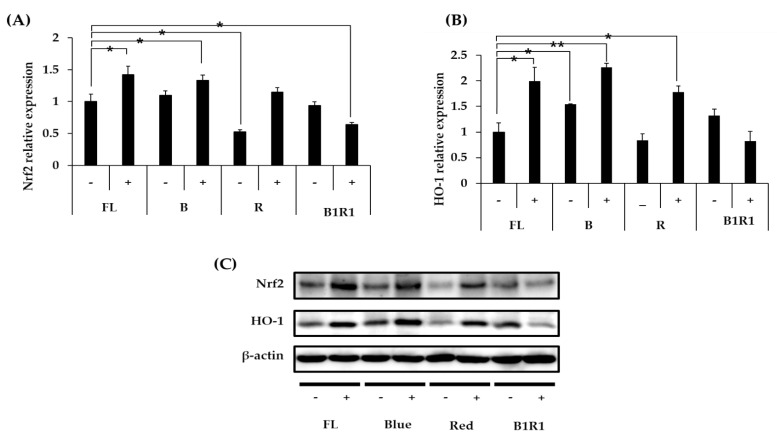
Effect of B. carinata microgreen extracts on the nuclear factor-erythroid 2-related factor (Nrf2) (**A**) and heme-oxygenase (HO-1) (**B**) protein expression in cells. Cells were treated with 100 µg/mL extracts for 48 h and the total expressed HO-1 protein and the Nrf2 were analyzed by Western blot (**C**) with β-Actin as the internal control. All data were presented as means ± standard error (*n* = 3. Student t-test was conducted to see the differences between relative expression of extracts from the control (fluorescence (FL) in absence of salt (−)) and extracts from treatment groups exposed to blue, red, B1R1 (blue plus red) in absence and presence (+) of salt, * = *p* < 0.05 and ** = *p* < 0.01 vs. the control.

We explored the antioxidative mechanisms of various extracts from cultivated microgreens, as well as their ability to activate the Nrf2 signaling pathway and up-regulate the HO-1 protein expression. Figure 5A–C shows that mainly *B carinata* microgreens rich in GLs activated the Nrf2 signaling pathway, causing an increase in the relative expression levels of HO-1 antioxidant protein. Extracts from salt-treated microgreens exposed to FL and B lights caused a significant increase in the expression of the Nrf2/HO-1 pathway, similar to extracts from microgreens exposed to R LED, which had a significant increase, particularly for HO-1 expression, compared to extracts from microgreens extracts from the control group. Salinity, on the other hand, decreased the relative expression levels of these antioxidants in the B1R1 LED exposed microgreens, with a significant decrease being observed for the Nrf2 (Figure 5A) relative expression but not for HO-1 (Figure 5B). Even though B1R1 LED exposed microgreens extracts had the least potential to activate the Nrf2/HO-1 system, we noticed a higher expression from the extracts from the microgreens in non-saline treatments of this LED, which had a higher total GLs content than their saline treatment counterparts.

### 3.8. Multivariate Analysis

**Figure 6:**
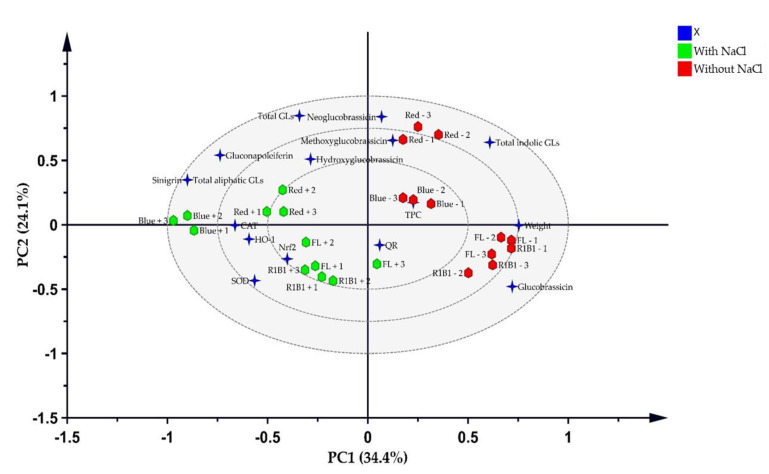
Partial least squares discriminant analysis (PLS-DA) score plots of variables and the treatment groups. The blue stars represent the variable and the green hexagons represent extracts from microgreens grown in the presence of salt stress while red hexagons are extracts from microgreens grown in absence of salt stress.

A PLS-DA (Figure 6) explains 58.5% of the total variation and shows a strong link between salt stress in the B and B1R1 LEDs with the accumulation of GLs compounds such as SIN the major compound and the different biological analysis evaluated was developed. The Hotelling T2, which is the critical limit in PCA and which displays the normality region corresponding to 95% confidence, showed a normal area with no serious outliers (Figure S2B). In the PCA bi-plot analysis, the groups were not clearly discriminated because the factors were over-lapped and this explained 65% of the total variation (Figure S2A). Thus, the variance on the X matrix was split by PLS-DA model into predictive and orthogonal variances and a supervised multivariate analysis showed the clear discrimination between the sample groups in the scores space (Figure 6) than PCA. These were separated into two clusters one group with salt and the other without salt. The regression line generated on the permutation test (Figure S3B) implied that the OPLS-DA model was run well. These results show that the model fit (*R*^2^) of X (*R*^2^*X*) of OPLS-DA was 0.687 indicating that over 65% of the variation could be modeled by the selected components. The important features in the data were considered to be six variables: weight (1.5), SIN (1.29), total aliphatic GLs (1.29), total indolic GLs (1.22), GNL (1.05), and GBS (1.01) (Figure S3A). CAT, SOD, Nrf2, HO-1, and weight showed a positive correlation with the saline-treated groups more than the non-saline treated groups while indolic GL compounds showed a similar tendency for both groups. In particular, biomass and GBS were strongly correlated with B1R1 and FL light. On the other hand, except for GBS, the rest of the GL compounds indicated that they were strongly influenced by R light regardless of the presence of salt.

## 4. Discussion

Plants and plant extracts in the *Brassicaceae* family are rich in diverse health-benefiting compounds such as phenolics and GLs compounds. Specifically, phenolic compounds derived from tannins and sinapic acid, flavonoids, such as quercetin and kaempferol [24] and GLs such SIN, GBS, GNBS, progoitrin, gluconapin, and gluconasturtiin [44] have been found in *B. carinata*. The profiles of these compounds have been shown to differ among *Brassicaceae* species due to plant-specific factors such as salinity and light [49] light. As a result, such compounds, that reflect the biological potential of plant-derived natural products are relevant targets for improving plants functionality.

Elicitors, activate chemical defense and various biosynthetic pathways in plants and are used during the cultivation process as a strategy to increase specific plant phytochemicals [19]. Although the use of salt as an elicitor has been shown to disrupt the plants’ osmotic balance and cause them to lose water to their surroundings [38], moderate salinity is especially effective in increasing compounds such as phenolic contents in brassica crops such as rapeseed [50], as seen in our results from the extracts of microgreens exposed to FL light and B LED. Comparable to our findings, salinity causes a decrease in weight, but the microgreens did not show any visible signs of stress, as previously observed in broccoli sprouts [38].

Light intensity and quality have a significant impact on plant growth and development, with the effects of these genotype-dependent factors causing different plant species to respond differently when exposed to specific wavelengths [29,51]. In this study, we found that microgreen biomass differed significantly after cultivating them under different light wavelengths, with the most noticeable differences on microgreens grown under B1R1, which had the highest biomass. However, our findings contradict those of a previous study in broccoli microgreens, in which B LED resulted in more shoots when compared to plants exposed to combined LEDs [37]. We speculate that R LED in our combination complemented the effects of B LED in increasing microgreens biomass, and given that we chose a different ratio from the previous study, it is also possible that this specific ratio had a positive influence on the biomass of microgreens plants.

Furthermore, light, which serves as an energy source for plants by influencing the photosynthetic light-dependent process, regulates the contents of some plant compounds such as phenolics and is used as an elicitor [29,52] as reported in broccoli microgreens [37], canola [29], lettuce [32] and Chinese mustard [52]. Several studies, however, have shown that light may also stress the plant during elicitation, resulting in photosynthesis suppression [53]. In our study, we found that B LED allowed for the accumulation of phenolic compounds while R LED did not. Previous studies indicate that B LED is a more powerful light source than R LED in terms of enhancing plant photosynthetic processes [37]. B LED influences plant leaf expansion, chlorophyll levels, light-induced stomatal opening, and photosynthesis [54], resulting in increased stomatal conductance, photo-synthetic electron transportability, and phenolic compounds [55,56].

Notably, previous studies have linked R light to increased starch levels in chloroplasts, which inhibits the translocation of photosynthates enzyme and ultimately reduces photosynthesis [57,58], possibly resulting in our observed lower phenolics in microgreens exposed to this LED. Moreover, the salt treatment of microgreen exposed R LED resulted in even lower phenolic contents due to the plants’ reduced ability to cope with both stresses. Although B1R1 LED exposure increased biomass, it did not affect phenolic compounds. Previous studies have shown that rapid metabolite changes do not occur primarily when exposed to a mixture of LEDs because the changes are more sensitive to changing specific monochromatic lights; thus, proper light ratio selection is required to achieve changes [32].

The negative ion mode was used for identification based on previous reports of its suitability in detecting DS-GLs compounds due to their structural properties [59]. The GLs deprotonated ions [M-H]^−^ (*m/z*), ion fragmentation patterns, and relative retention times of the compounds compared with that of available standards aids in the identification of the distinct compounds. We found out that the fragmentation of GLs revealed two groups of specific fragments. The MS^2^ fragment corresponds to the aglycone portion and produces stable ions at *m/z* 195, 275, and 259 (by loss of R1-N=C=O, together with R1-N=C=S from the deprotonated molecule [M-H]^−^), whereas *m/z* 241 ions are formed by the cleavage of water (H_2_O, 18 Da). Fragmentation of the primary ions at *m/z* 259 resulted in another fragment that corresponds to the D-thioglucose group [C_6_H_11_O_5_S]^−^; which is shown to originate from a rearrangement reaction where the sulfate group is transferred to the thioglucosidase side chain in earlier studies [60].

Except for GNL, the other five compounds found in *B carinata* had previously been reported; however, we did not find progoitrin, gluconapin, or gluconasturtiin, which have also been reported in the plant [44]. The total aliphatic GLs content was consistently high in all treatments, owing to increasing levels of SIN, the most abundant compound in *B. carinata* [2]. Salinity stress significantly increased aliphatic GLs contents, which is consistent with previous research among *Brassicaceae* vegetables [22,61,62], and exposure to light wavelengths may have produced a synergistic reaction, leading to an even greater increase. In particular, B LED produced the highest SIN levels in microgreens, although in contrast to R LED, which increased SIN in canola [29] and broccoli microgreens [63]. These findings point to the possibility of a species-dependent metabolite increase as a result of light exposure. In addition, the decrease in GBS seen in the microgreens irradiated to B and R LEDs is in agreement with other studies involving elicitor treatments where GBS is converted to its byproducts [64]. The differences observed in the accumulation of GBS byproducts under various LED treatments, in saline and non-saline conditions are attributable to changes in the expression of several genes involved in their biosynthesis [64]. From our findings, exposure to R LED is likely to have caused the expression of more genes involved in indole GLs biosynthesis leading to the conversion of GBS to most of its products (HGBS, MGBS), whereas exposure to B LED was biased toward one arm of the biosynthetic pathway where GBS is converted to GNBS as seen in other studies involving plants cultivated in non-saline conditions [65].

The production of GLs in salt-treated plants is believed to protect plants from abiotic stress, and the salt concentration used in this study was within the salinity tolerance threshold [66] to activate genes involved in the biosynthesis of these aliphatic GLs in *B. carinata* microgreens, as expected. This is consistent with findings from other studies in which a salt concentration of 100 mM significantly increased the nutritional value of *Brassicaceae* plants such as radish sprouts [23], broccoli sprouts [38], and broccoli seedlings [61], demonstrating its utility in increasing the content of these health-promoting compounds [23,67].

Generally, cruciferous vegetables are consumed for their health-promoting benefits, including antioxidant, anti-proliferative, and chemo-preventive activities contributed mainly by GLs and phenolics [24,68,69,70]. Currently, there are growing pieces of evidence suggesting that oxidative stress from reactive oxidants plays a key role in the pathogenesis of several diseases [11]. In recent decades, multiple strategies were evaluated to reduce the impact of oxidative stresses including the use of naturally produced enzymes such as SOD and CAT as well as their overexpression by other compounds. Several pieces of literature have cited the importance of dietary polyphenols and the great roles they play in activating and up-regulating the expression of antioxidant enzymes including SOD, CAT, glutathione reductases, glutathione peroxidases and glutathione-*s*-transferases. An increase in the activities of these antioxidant enzymes in cells induced with ROS proves the enzymes’ well adaptation in maintaining a balance of the reactive oxidants in the cells, therefore lowering oxidative stress [71].

The antioxidant enzymes are the first defense system of a cell against ROS [72]. The collapse of a balance in the production of reactive oxidants and scavenging ability causes an accumulation of ROS such as superoxides (O_2_^−^) and hydrogen peroxides (H_2_O_2)_ which result in cell death or proliferation through DNA damage, protein oxidation, and lipid peroxidation [73]. In the cellular systems, antioxidant enzymes help to maintain this balance. For instance, SOD acts to convert the O_2_^−^ radicals through dismutation to less toxic, H_2_O_2_ while CAT eliminates the H_2_O_2_ and changes it to water and oxygen [74]. Increased enzyme activity in in vitro assay is associated with reduced oxidative stress and reduced cell damage. Our observed increased antioxidant enzyme activity in cells treated with the microgreens extracts was facilitated by the available contents of natural antioxidants such as polyphenols and GLs as reported in other studies [71,75].

Our investigation on the mechanism of bioactive compounds in *B. carinata* microgreen also displayed an enhancement of Nrf2 and HO-1 expression. This enhancement is essential for cell protection due to the critical roles the Nrf2 pathway plays in reducing inflammation and oxidative stress. Plant compounds such as GSHPs activate the Nrf2/HO-1 antioxidant pathway and are therefore suitable as protective and treatment agents for oxidative stress, inflammatory diseases [76]. Several researchers are interested in identifying activators of this pathway as potential therapeutic strategies for various diseases including cancer [77,78]. Cruciferous vegetables have been identified as valuable sources of GSHPs, which activate Nrf2 and thus up-regulate phase II enzymes as well as antioxidant enzymes such as HO-1 [76]. In our study, microgreens cultivated in saline conditions under B, R, and FL light especially, accumulated the highest contents of GLs which possibly contributed to the pathway activation and resulted in adding the therapeutic value of microgreens.

## 5. Conclusions

In this study, we investigated whether *B carinata* is suited for the indoor farming system and whether we can achieve proper cultivation conditions through elicitation, for the production of microgreens with high biomass, rich in phytochemicals, and improved biological potential including antioxidant activities. Salinity and B LED were found to be important in promoting GLs and phenolics in *B. carinata* microgreens, as well as their overall biological activity, whereas R LED was a stressed condition that reduced the plants’ ability to photosynthesize, resulting in low accumulation of beneficial phytochemicals, particularly in saline conditions. Furthermore, non-saline conditions with B1R1 LED were comparable to the control FL condition for producing microgreens with increased biomass. To increase and preserve the beneficial metabolite content and nutritional value of *B carinata*, B LED supplemental wavelength and proper blue plus red LED ratios may be beneficial in microgreens cultivation in the vertical farming system.

## Figures and Tables

**Table 1:** Desulfo glucosinolates determined through ultra-high performance-quadrupole time of flight mass spectrometry (UPLC QTOF-MS).

No.	Compound	Abbreviation	RT (min)	Formula	Precursor ion	Production	Relative Response Factor
1	Sinigrin	SIN	5.51	C_10_H_17_NO_6_S	278.9960	195.0396 116.0270	1.05
2	Gluconapoleiferin	GNL	6.72	C_12_H_21_NO_10_S_2_	322.1013	195.0378 130.0408	1.05
3	4-Hydroxy glucobrassicin	HGBS	7.17	C_16_H_20_N_2_O_10_S_2_	383.1020	221.0448 195.0394	0.29
4	Glucotropaeolin	GTR	8.31	C_14_H_19_NO_9_S_2_	328.0976	195.0421 166.0406	1.00
5	Glucobrassicin	GBS	9.33	C_16_H_20_N_2_O_9_S_2_	367.1112	205.0524 195.0408	0.31
6	4-Methoxy glucobrassicin	MGBS	11.25	C_17_H_22_N_2_O_10_S_2_	397.1167	235.0615 195.0389	0.26
7	Neoglucobrassicin	GNBS	12.44	C_17_H_22_N_2_O_10_S_2_	397.1228	235.0633 195.0410	0.21

## Data Availability

The data presented in this study are available in article and sup-plementary material.

## Supplementary Materials

The following are available online at https://www.mdpi.com/article/10.3390/antiox10081183/s1, Figure S1: UPLC-DAD chromatogram of available standards detected at 229 nm, Figure S2: PCA bi-plot of *B.carinata* microgreens (**A**) showing separation of groups on the space. Green hexagons represent the group of microgreen samples under different lights that were treated to salt stress. Red hexagons: represent the group of microgreen samples that were not treated with salt. Blue stars: X-variable. (**B**) Hotelling’s T2 range plots of the PCA belong to the normal area, Figure S3: Multivariate statistical analysis done by SIMCA program of *B. carinata* microgreens. (**A**) The VIP score of data. (**B**) showing a validation plot for OPLS-DA. Green circle: *R*^2^*Y* (Cum), Blue pentagons: *Q*^2^ (Cum).
