# Supplementary material for: Exposure to Salinity and Light Spectra Regulates Glucosinolates, Phenolics, and Antioxidant Capacity of Brassica carinata L. Microgreens
Source: Antioxidants (Basel). 2021 Jul 26;10(8):1183. doi: 10.3390/antiox10081183 (PMC8389028; doi:10.3390/antiox10081183)
Supplement: Supplementary file 1 [file antioxidants-10-01183-s001.zip › antioxidants-1300785-supplementary.pdf]

Supplementary data:

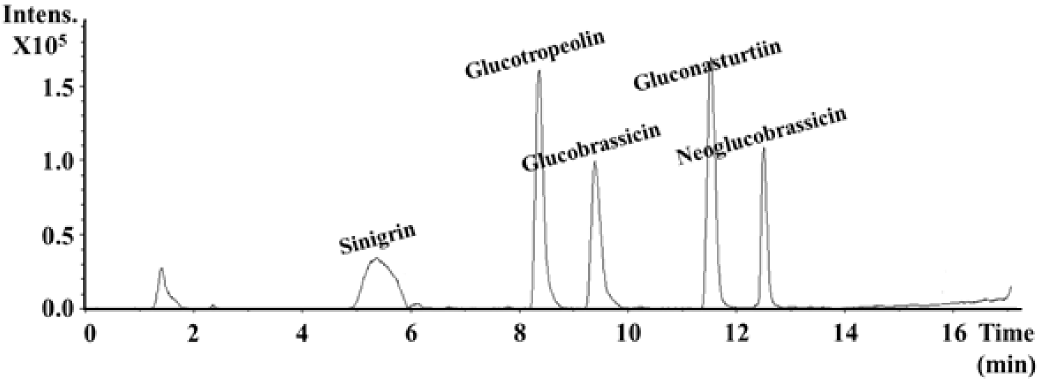

Figure S1. UPLC-DAD chromatogram of available standards detected at 229 nm.

A B

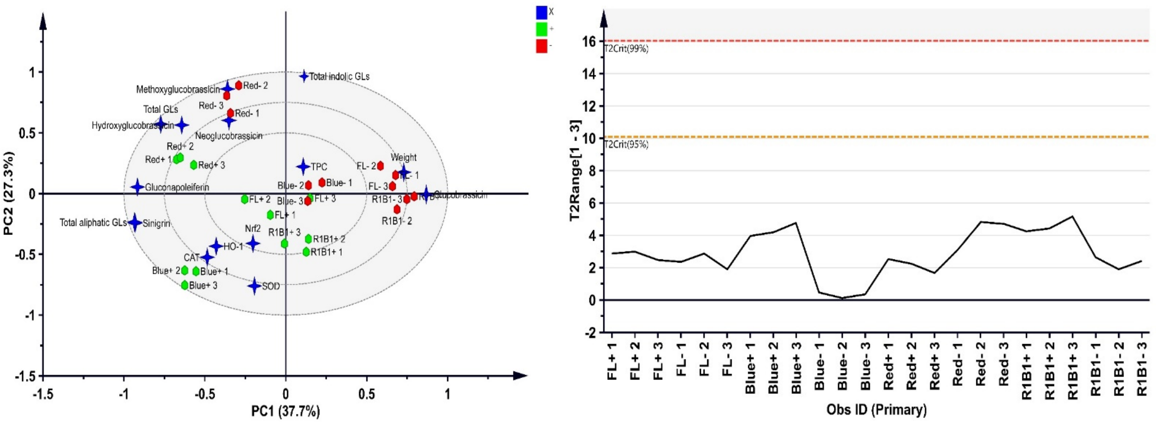

Figure S2: PCA bi-plot of *B.carinata* microgreens (A) showing separation of groups on the space. Green hexagons represent the group of microgreen samples under different lights that were treated to salt stress. Red hexagons: represent the group of microgreen samples that were not treated with salt. Blue stars: X-variable. (B) Hotelling's T2 range plots of the PCA belong to the normal area.

A B

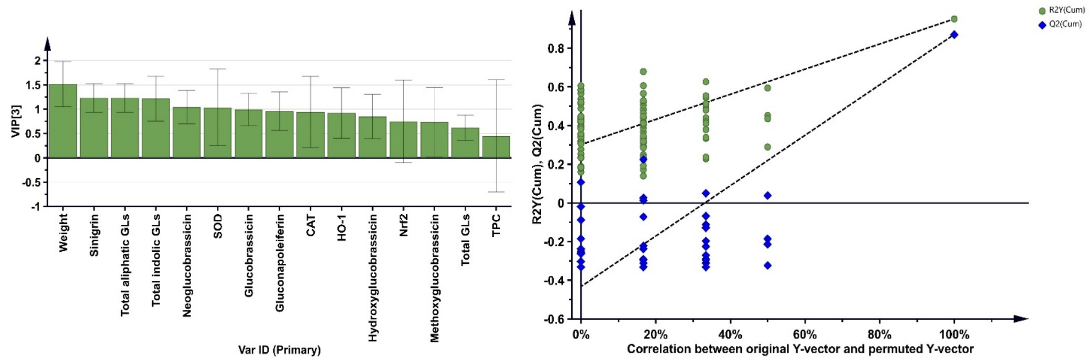

Figure S3: Multivariate statistical analysis done by SIMCA program of B. carinata microgreens. (A)The VIP score of the different variables. (B) showing a validation plot for OPLS-DA. Green circle: R2Y (Cum), Blue pentagons: Q2 (Cum).
